# *saeRS* and *sarA* Act Synergistically to Repress Protease Production and Promote Biofilm Formation in *Staphylococcus aureus*

**DOI:** 10.1371/journal.pone.0038453

**Published:** 2012-06-07

**Authors:** Lara N. Mrak, Agnieszka K. Zielinska, Karen E. Beenken, Ian N. Mrak, Danielle N. Atwood, Linda M. Griffin, Chia Y. Lee, Mark S. Smeltzer

**Affiliations:** Department of Microbiology and Immunology, University of Arkansas for Medical Sciences, Little Rock, Arkansas, United States of America; University of Illinois at Chicago College of Medicine, United States of America

## Abstract

Mutation of the staphylococcal accessory regulator (*sarA*) limits biofilm formation in diverse strains of *Staphylococcus aureus*, but there are exceptions. One of these is the commonly studied strain Newman. This strain has two defects of potential relevance, the first being mutations that preclude anchoring of the fibronectin-binding proteins FnbA and FnbB to the cell wall, and the second being a point mutation in *saeS* that results in constitutive activation of the *saePQRS* regulatory system. We repaired these defects to determine whether either plays a role in biofilm formation and, if so, whether this could account for the reduced impact of *sarA* in Newman. Restoration of surface-anchored FnbA enhanced biofilm formation, but mutation of *sarA* in this *fnbA*-positive strain increased rather than decreased biofilm formation. Mutation of *sarA* in an *saeS*-repaired derivative of Newman (P18L) or a Newman *saeRS* mutant (Δ*saeRS*) resulted in a biofilm-deficient phenotype like that observed in clinical isolates, even in the absence of surface-anchored FnbA. These phenotypes were correlated with increased production of extracellular proteases and decreased accumulation of FnbA and/or Spa in the P18L and Δ*saeRS sarA* mutants by comparison to the Newman *sarA* mutant. The reduced accumulation of Spa was reversed by mutation of the gene encoding aureolysin, while the reduced accumulation of FnbA was reversed by mutation of the *sspABC* operon. These results demonstrate that *saeRS* and *sarA* act synergistically to repress the production of extracellular proteases that would otherwise limit accumulation of critical proteins that contribute to biofilm formation, with constitutive activation of *saeRS* limiting protease production, even in a *sarA* mutant, to a degree that can be correlated with increased enhanced capacity to form a biofilm. Although it remains unclear whether these effects are mediated directly or indirectly, studies done with an *sspA::lux* reporter suggest they are mediated at a transcriptional level.

## Introduction

*Staphylococcus aureus* is capable of causing diverse forms of human infection. Understanding the pathogenesis of these infections is complicated by the diversity among clinical isolates of *S. aureus*, and this makes it imperative to understand the impact of this diversity on clinically relevant phenotypes. Two of the most important of these phenotypes are toxin production and biofilm formation, with the former being a defining characteristic of acute infections and the latter being a defining characteristic of chronic infections [1]. We have a specific interest in chronic orthopaedic infections, and in this context we have placed a primary emphasis on biofilm formation as a contributing factor to the therapeutic recalcitrance of these infections to conventional antimicrobial therapy [2]. The therapeutic outcome in such infections is often poor irrespective of the antibiotic resistance status of the offending strain [3].

We demonstrated that mutation of the staphylococcal accessory regulator (*sarA*) limits biofilm formation in genotypically and phenotypically diverse clinical isolates of *S. aureus* to a degree that can be correlated with increased antibiotic susceptibility under both *in vitro* and *in vivo* conditions [4], [5]. This suggests that inhibitors of *sarA* expression and/or function could be used to therapeutic advantage. However, the efficacy of such inhibitors could be compromised by two experimental observations made during the course of these studies. The first is that in some strains mutation of *sarA* has also been associated with increased production of alpha toxin [6], an important virulence factor in many forms of *S. aureus* infection, including those caused by isolates of the USA300 clonal lineage [7]. To address this issue, we explored the mechanistic basis for the strain-dependent impact of *sarA* on toxin production, and the results led us to conclude that, with few exceptions, mutation of *sarA* results in reduced accumulation of critical extracellular toxins, including alpha toxin and phenol soluble modulins (PSMs), at least as assessed under *in vitro* conditions [8]. One of the exceptions is the commonly studied strain Newman, which is characterized by a point mutation that results in constitutive activation of the *saePQRS* regulatory system [9], and we confirmed that this accounts for the apparent increase in the production of both alpha toxin and PSMs in a Newman *sarA* mutant, owing to the limiting impact of *saeRQRS* on the production of extracellular proteases [8].

The second potentially compromising factor is that the impact of mutating *sarA* on biofilm formation is also strain-dependent, with Newman once again being a primary example. This is potentially relevant in that we have also demonstrated that the increased production of extracellular proteases plays an important role in defining the biofilm-deficient phenotype of *S. aureus sarA* mutants [10], [11]. Based on these observations, it would be anticipated that Newman would have an enhanced capacity to form a biofilm owing to its reduced production of extracellular proteases, but we have found that this is not the case [12]. However, the biofilm phenotype of Newman is further complicated in that *fnbA* and *fnbB*, which encode fibronectin-binding proteins (FnbA and FnbB), which are known to contribute to biofilm formation in *S. aureus* [13], [14], have nonsense mutations that result in the production of truncated proteins that cannot be anchored to the cell surface [15].

These two defects are interrelated in that, like *sarA*, *saeRS* enhances transcription of *fnbA* as well as other surface-associated binding proteins [16]. Thus, one possible explanation for the biofilm-deficient phenotype of *sarA* mutants is the reduced production of surface-associated proteins such as FnbA. However, several reports have suggested that the reduced capacity of *S. aureus sarA* mutants to bind fibronectin is defined by the increased production of extracellular proteases rather than transcriptional changes in expression of the *fnbA* or *fnbB* genes [6], [17]. Thus, both *saeRS* and *sarA* impact the production of adhesins known to contribute to biofilm formation [16] and proteases known to limit the accumulation of these adhesins. The fact that Newman is lacking surface-anchored FnbA therefore raises the possibility that the reduced capacity of Newman to form a biofilm, and the reduced impact of *sarA* on biofilm formation, are both due to the reduced availability of a critical surface-associated target of extracellular proteases.

To investigate this, we restored the ability of Newman to produce surface-associated FnbA and examined the impact on biofilm as a function of *sarA*. While this did enhance biofilm formation, it also reversed the biofilm-deficient phenotype of the isogenic *sarA* mutant, with the *fnbA*-positive Newman *sarA* mutant exhibiting an enhanced capacity to form a biofilm. Subsequent studies demonstrated that this is due to constitutive activation *saeRS,* resulting in reduced production of extracellular proteases and consequently increased accumulation of both FnbA and protein A (Spa).

## Results

**Figure 1:**
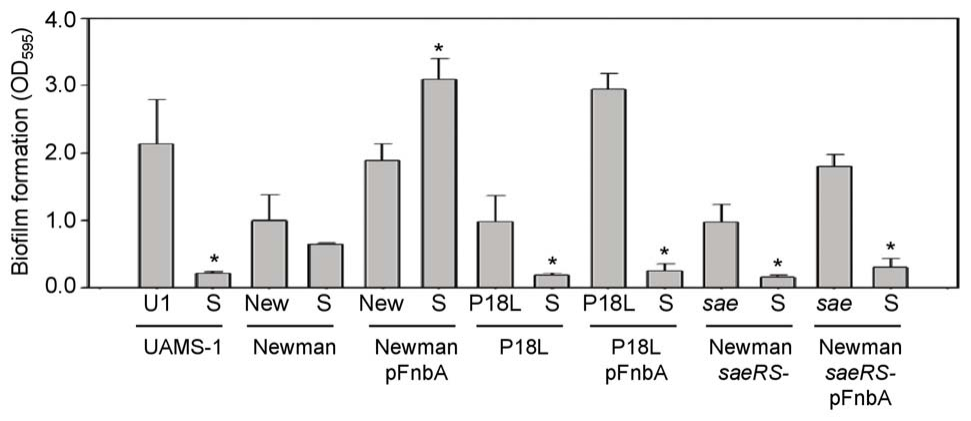
Impact of *saeRS* and surface-associated FnbA on biofilm formation in Newman. Surface-anchored FnbA was restored in Newman (New), its *saeS*-repaired derivative (P18L), and its isogenic *saeRS* mutant (*sae*) by introduction of a plasmid-borne copy of *fnbA*. Biofilm formation was assessed using a microtiter plate assay, with UAMS-1 (U1) and its *sarA* mutant included as positive and negative controls, respectively. *sarA* mutants are designated as “S.” Asterisks indicate statistical significance (p<0.05) by comparison to the isogenic parent strain (WT).

As in our previous studies [12], Newman was found to have a reduced capacity to form a biofilm by comparison to the clinical isolate UAMS-1, and mutation of *sarA* resulted in only a modest decrease in biofilm formation (Fig. 1). Introduction of an intact copy of *fnbA* on a plasmid (pFnbA) increased biofilm formation in Newman to levels that approached those observed with UAMS-1, suggesting that the inability to anchor FnbA to the cell surface contributes to the reduced capacity of Newman to form a biofilm. This effect was also apparent in a derivative of Newman in which the *saeS* defect was repaired (P18L), but it was moderated in an isogenic *saeRS* mutant, a phenotype that is consistent with the demonstration that activation of *saeRS* enhances transcription of *fnbA* [16]. More importantly, mutation of *sarA* in the pFnbA derivative of Newman resulted in an increased rather than decreased capacity to form a biofilm (Fig. 1). In contrast, mutation of *sarA* in both the P18L pFnbA derivative and the pFnbA *saeRS* mutant limited biofilm formation to a degree comparable to that observed in a UAMS-1 *sarA* mutant (Fig. 1). However, this was also true in *sarA* mutants generated in these strains in the absence of pFnbA, thus suggesting that the disparate *sarA*-dependent biofilm phenotypes observed in Newman vs. its *saeRS* derivatives involve something other than the impact of *saeRS* on the production of surface-associated FnbA.

**Figure 2:**
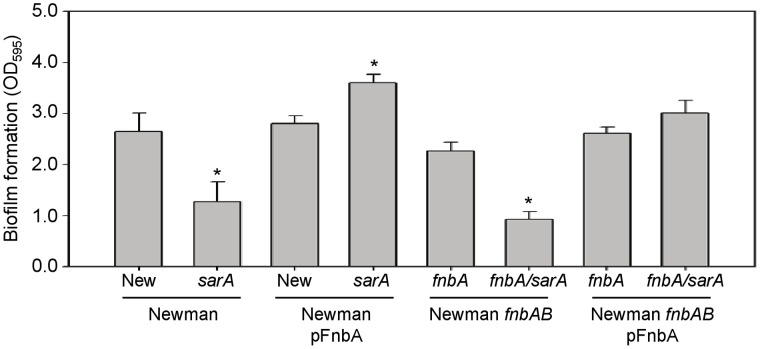
Impact of endogenous fibronectin-binding proteins on biofilm formation in Newman. Biofilm formation was assessed using a microtiter plate assay in Newman with and without introduction of surface-anchored FnbA (pFnbA) and/or mutation of its endogenous *fnbA* and *fnbB*. Asterisks indicate statistical significance (p<0.05) by comparison to the isogenic parent strain (WT).

Newman encodes both *fnbA* and *fnbB*, with the defect in these genes precluding anchoring of the corresponding proteins to the cell surface but not their production [15]. This raises the possibility that the increased production of extracellular forms of these proteins impact the *sarA*-dependent biofilm phenotype. This is particularly true since protein A has been shown to promote biofilm formation in both its surface associated and extracellular forms [18]. To investigate this, we generated *fnbA/fnbB* mutants in Newman, its *sarA* mutant, and their pFnbA derivatives and assessed the impact on biofilm formation, but this had little impact on biofilm phenotype of the Newman pFnbA *sarA* mutant (Fig. 2). This provides further support for the hypothesis that these disparate phenotypes are due to something other than the impact of *saeRS* on the transcription of *fnbA*.

**Figure 3:**
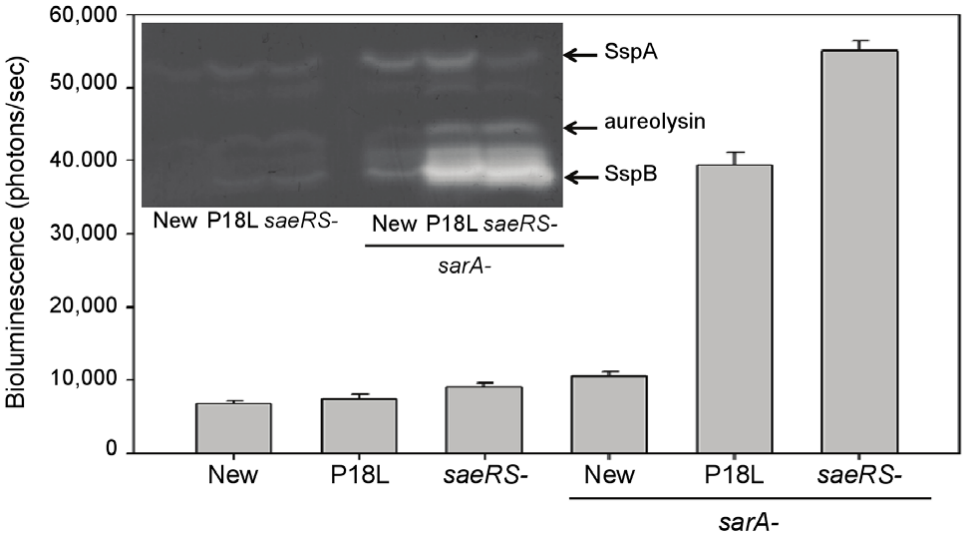
Impact of *saeRS* and *sarA* on protease production. Production of extracellular proteases in derivatives of Newman as a function of *saeRS* and *sarA* was assessed by zymography using gelatin as the substrate. The presumed identity of individual proteases is indicated to the right. The graph illustrates relative expression levels the *sspA* promoter as assessed using an *sspA::lux* reporter. Differences between the Newman *sarA* mutant, the P18L *sarA* mutant, and the *saeRS/sarA* mutant were all statistically significant (p<0.05) by comparison to Newman. Differences between the *sarA/saeRS* and the P18L *sarA* mutants, and between the P18L *sarA* mutant and the Newman *sarA* mutant, were also significant.

When we examined the production of extracellular proteases in Newman and its *saeRS* and *sarA* derivatives, we found a direct correlation between the production of these proteases and the functional status of both *saeRS* and *sarA*. Specifically, protease production was lowest in Newman and increased progressively as the relative activity of both *saeRS* and *sarA* declined (Fig. 3). Most importantly, while mutation of *sarA* resulted in increased production of multiple extracellular proteases in all strains, this effect was moderated in a Newman *sarA* mutant. This was also evident in reporter assays using an *sspA::luxABCDE* reporter, suggesting that these changes occur at the transcriptional level.

**Figure 4:**
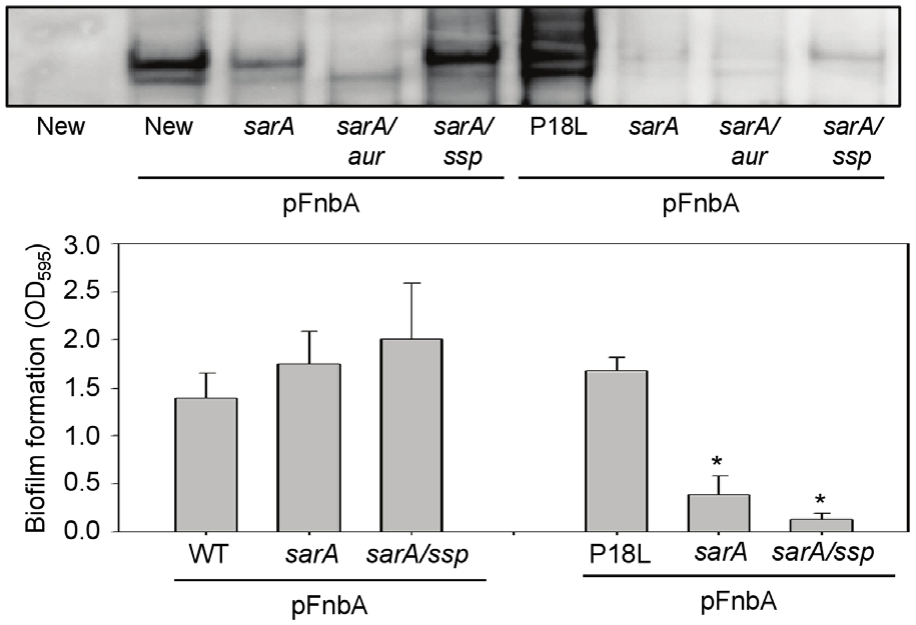
Impact of *sarA*, *saeRS*, and extracellular proteases on accumulation of FnbA and biofilm formation. Top: Relative amounts of surface-anchored FnbA were assessed in Newman (New), its *saeS*-repaired derivative (P18L), and its *saeRS* mutant (*sae*) after introduction of an intact copy of *fnbA* on a plasmid. Newman without this plasmid was included as a negative control. The impact of mutating *sarA* was assessed in each of these strains together with the impact of mutating the gene encoding aureolysin (*aur*), *sspABC* (*ssp*) or *sae* on the phenotype of the *sarA* mutants. Bottom: Biofilm formation was assessed by microtiter plate assay in Newman and P18L as well as their *sarA* and *sarA/ssp* derivatives after the introduction of pFnbA.

When we examined the accumulation of surface-associated FnbA, we found that it was present in reduced amounts in the pFnbA Newman *sarA* mutant by comparison to pFnbA Newman, and that this effect was reversed by mutation of *sspABC* (Fig. 4). In contrast, mutation of the gene encoding aureolysin (*aur*) had little impact on the FnbA phenotype of the Newman *sarA* mutant. Surface-associated FnbA was also detected in pFnbA P18L, but it was reduced to almost undetectable levels in the isogenic *sarA* mutant, and concomitant mutation of *sspABC* had relatively little impact. This was surprising in that production of both SspA and SspB was higher in a P18L *sarA* mutant than a Newman *sarA* mutant (Fig. 3), thus suggesting that mutation of *sspABC* would have a greater impact on the accumulation of FnbA in the P18L *sarA* mutant. Nevertheless, these same relative levels of FnbA production were evident in the context of biofilm formation, with mutation of *sspABC* enhancing biofilm formation in a pFnbA Newman *sarA* mutant, albeit to a modest extent, but having no impact on biofilm formation in the pFnbA P18L *sarA* mutant (Fig. 4). This suggests that, while surface associated FnbA is important, some other difference(s) must also exist between these strains that is (are) both relevant to biofilm formation and moderated in an *saeRS*-dependent manner.

**Figure 5:**
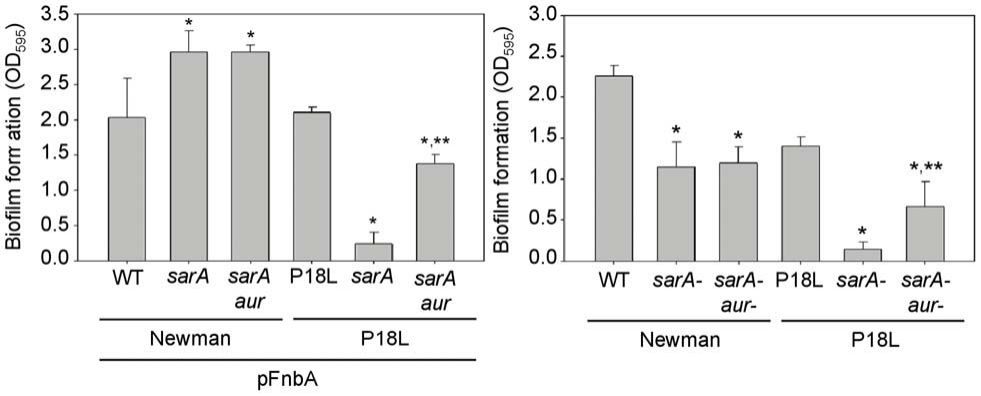
Impact of aureolysin on *saeRS* and *sarA*-dependent biofilm formation. Biofilm formation was assessed in Newman, its P18L derivative, and their *sarA*, *sarA/aur* and *sarA/ssp* mutants with (left) and without (right) the introduction of an intact copy of *fnbA*. A single asterisk indicates statistical significance (p<0.05) by comparison to the isogenic parent strain, while the double asterisk indicates statistical significance (p<0.05) by comparison to the isogenic *sarA* mutant.

Mutation of *aur* enhanced biofilm formation in a P18L *sarA* mutant, but had no impact on biofilm formation in a Newman *sarA* mutant, and this was true irrespective of the presence of pFnbA (Fig. 5). However, the lack of a phenotype in the pFnbA Newman *sarA/aur* mutant must be taken in context in that biofilm formation was already elevated in the isogenic pFnbA Newman *sarA* mutant, meaning biofilm formation in this strain may be at a maximum defined by this assay. However, the observation that these same disparate *sarA/aur* phenotypes were apparent in the absence of pFnbA gene (Fig. 5) confirms the existence of an *saeRS*-dependent biofilm phenotype in *S. aureus* that cannot be explained by the impact of proteases on the accumulation of surface associated FnbA.

**Figure 6:**
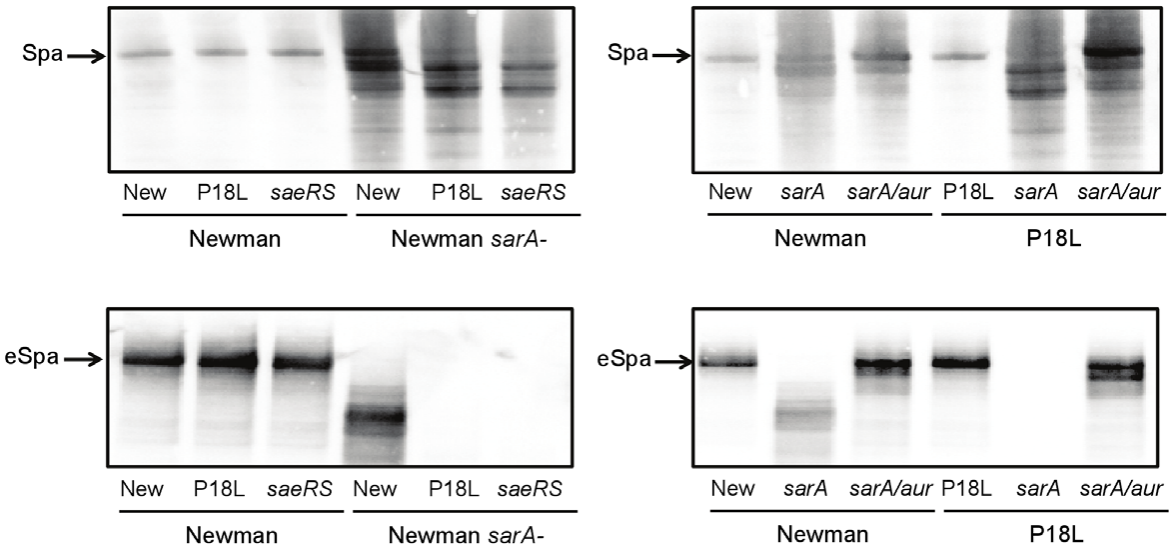
Impact of *saeRS* and *sarA* on the abundance of protein A (Spa). The abundance of surface associated (top) and extracellular Spa (bottom) was assessed by western blot using anti-Spa antibody. Strains include Newman (WT), its *saeS*-repaired derivative (P18L), its isogenic *saeRS* mutant, and derivatives of each in which *sarA* was mutated alone or in combination with *aur*.

Mutation of *saeRS* or *sarA* has also been associated with reduced production of Spa, and this has been attributed to transcriptional changes [19]. However, like FnbA, the production of extracellular proteases has been shown to limit the accumulation of Spa [17]. The production of Spa in both its surface-associated and extracellular forms (eSpa) has also been correlated with an enhanced capacity to form a biofilm [18]. Based on these considerations, we examined the relative levels of surface-associated and eSpa in Newman and all of its *saeRS* and *sarA* derivatives. The amounts of both were comparable in Newman, its P18L derivative, and its isogenic *saeRS* mutant (Fig. 6). While indirect, this suggests that *saeRS* has relatively little impact on *spa* transcription. In contrast, the amount of both surface-associated and eSpa was decreased in a Newman *sarA* mutant, but decreased even further in the isogenic P18L *sarA* and *saeRS/sarA* mutants (Fig. 6), corresponding with biofilm formation (Fig. 1). The fact that this was protease mediated was confirmed by demonstrating that concomitant mutation of *aur* reversed this phenotype (Fig. 6).

**Figure 7:**
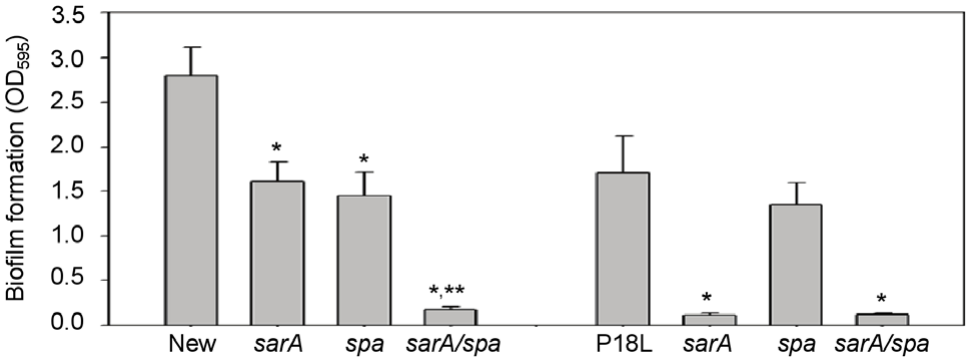
Impact of protein A on biofilm formation in Newman. Biofilm formation was assessed using a microtiter plate assay in Newman and its *sarA* and *spa* derivatives without the introduction of surface-anchored FnbA. Single asterisks indicate statistical significance (p<0.05) by comparison to the isogenic parent strain. Double asterisk indicates significance by comparison to the isogenic *sarA* mutant.

Thus, one explanation for the increase in biofilm formation in a pFnbA Newman *sarA* mutant is the relatively high availability of FnbA and Spa by comparison to P18L *sarA* and *saeRS/sarA* mutants, resulting in an enhanced capacity to form a biofilm in the former and a biofilm-deficient phenotype in the latter. In a pFnbA Newman *sarA* mutant, this would be presumably be due to both increased transcription of *fnbA* [16] and decreased degradation of the resulting protein. If this is true, then it would be anticipated that, in the absence of pFnbA, mutation of *spa* in a Newman *sarA* mutant would limit biofilm formation to a degree comparable to that observed in a P18L *sarA* mutant, and we found that this was in fact the case (Fig. 7).

**Figure 8:**
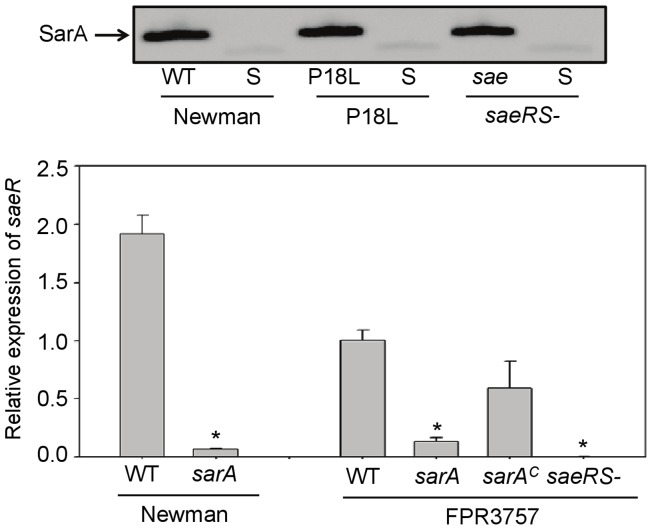
Interactions between *sarA* and *saeRS*. Top: Production of SarA was assessed by western blot using SarA antibody in the indicated strains (WT) and their isogenic *sarA* mtuants (S). Bottom: Impact of *sarA* on transcription of *saeR* in post-exponential cultures (OD_560_ = 3.0) was assessed by qRT-PCR. Results are shown relative to those observed with FPR3757, which were set to a value of 1.0. Asterisks indicate statistical significance (p<0.05) by comparison to the parent strain.

**Figure 9:**
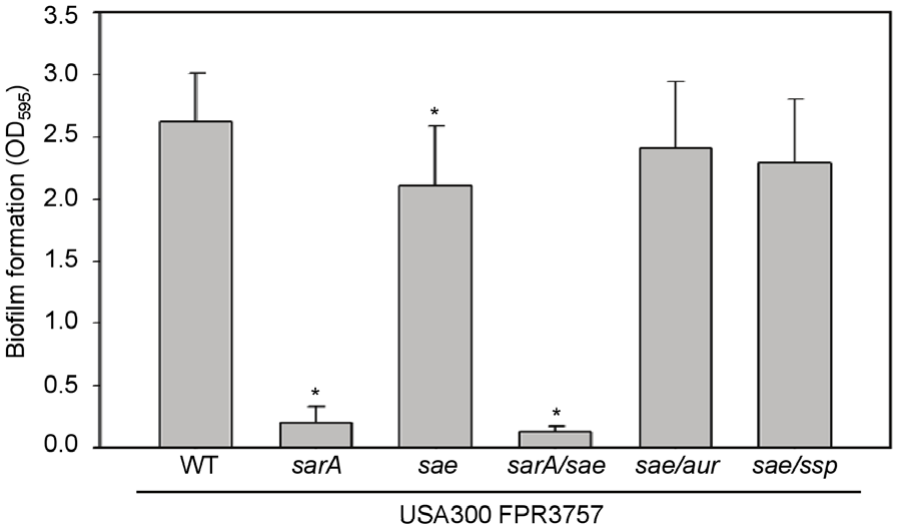
Impact of *saeRS* and *sarA* on biofilm formation in clinical isolates. Biofilm formation was assessed in USA300 strain FPR3757 and its isogenic *sarA* and *saeRS* (*sae*) mutants. A single asterisk indicates statistical significance (p<0.05) by comparison to the isogenic parent strain. Differences between the FPR3757 *saeRS* mutant and the *saeRS/aur* and *saeRS/ssp* mutants were not significant.

Finally, we investigated the interaction between *sarA* and *saeRS* by examining the impact of mutating one on the other. The relative activity of *saeRS* had no impact on the production of SarA, but mutation of *sarA* resulted in reduced transcription of *saeRS* even in the context of the otherwise constitutive activation of *saeRS* in Newman (Fig. 8). However, even with decreased, but not elimination of, *saeR* transcription, constitutive activation of the *saePQRS* operon can be achieved by constitutive phosphorylation of SaeR by SaeS. This transcriptional downregulation of *saeR* by SarA was true in the USA300 isolate FPR3757 as well. Moreover, mutation of *saeRS* in FPR3757 was correlated with a reduced capacity to form a biofilm (Fig. 9). Importantly, while this biofilm-deficient phenotype was not apparent in a comparison of pFnbA Newman and its pFnbA *saeRS* mutant, it was apparent in a comparison of pFnbA P18L and the pFnbA *saeRS* mutant, in which the functional status of *saeRS* and *fnbA* are similar to FPR3757 and its isogenic *saeRS* mutant (Fig. 1). While the biofilm defect in the FPR3757 *saeRS* mutant was modest, particularly by comparison to mutation of *sarA*, it was nevertheless statistically significant, and this phenotype could be “complemented” by mutation the genes encoding specific extracellular proteases (Fig. 9). This, along with the observation that mutation of *saeRS* has no effect on biofilm formation in a FPR3757 *sarA* mutant, suggests that mutation of *saeRS* resulting in inactivation would not jeopardize therapy with a *sarA* inhibitor. Furthermore, these results further demonstrate the correlation between reduced *saeRS* expression, increased protease production, and a reduced capacity to form a biofilm, and these correlations are independent of, but synergistic with, the impact of *sarA* on these same phenotypes.

## Discussion

The *saePQRS* regulatory system has been implicated in biofilm formation in both *S. epidermidis* and *S. aureus* [20], [21]. In *S. epidermidis*, mutation of *saeRS* enhances biofilm formation, and this has been correlated with increased autolysis and the increased availability of extracellular DNA [21]. In contrast, the only study examining the impact of *saeRS* on biofilm formation in *S. aureus*, which was also done with Newman, found that mutation of *saeRS* resulted in a reduced capacity to form a biofilm [20]. In fact, mutation of *saeRS* limited biofilm formation in this study to a degree that exceeded even that observed with the isogenic *sarA* mutant [20]. This was attributed to reduced transcription of the *ica* operon and the genes encoding the secreted proteins Emp and Eap [20].

We were unable to reproduce this phenotype using our assay conditions. Specifically, Newman, its P18L derivative, and its isogenic *saeRS* mutant exhibited a comparable capacity to form a biofilm that significantly exceeded that observed with the isogenic Newman *sarA* mutant. However, there are two potentially important experimental differences that could explain this discrepancy. First, the earlier study focused on biofilm formation under iron-limited conditions [20], which we did not address in our experiments. The second and potentially more important, particularly in the context of extracellular proteases, is that our *in vitro* biofilm assays employed a substrate coated with human plasma proteins. We do this for three reasons, the first being that implanted medical devices are rapidly coated with plasma proteins. The second is that coating the substrate with human plasma significantly enhances biofilm formation in genotypically and phenotypically diverse strains of *S. aureus* [12]. The third is that, with the exception of extracellular nucleases [22], the results we have observed in all of our *in vitro* biofilm assays have been consistent with those we have observed under *in vivo* conditions. Most importantly, this is true in the context of *sarA*, which we have demonstrated results in a reduced capacity to form a biofilm to a degree that can be correlated with increased antibiotic susceptibility under both *in vitro* and *in vivo* conditions [4], [5].

**Figure 10:**
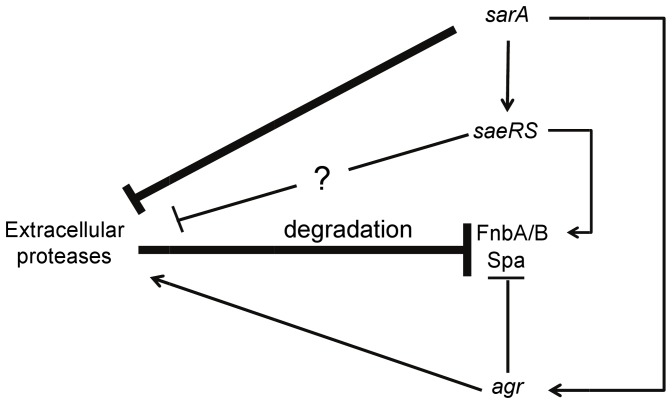
Model for the synergistic impact of *saeRS* and *sarA* on biofilm formation. Both *sarA* and *saeRS* repress the production of extracellular proteases, with *sarA* having the greater effect owing to both direct repression and activation of *saeRS* transcription. This repression relieves the protease-mediated “repression” of specific surface proteins arising from degradation. This in turn promotes accumulation of these proteins and an enhanced capacity to form a biofilm. The accessory gene regulator (*agr*) has the opposite effects on all of these phenotypes, but, as previously described, the impact of *sarA* occurs independently of *agr*, and *sarA* is epistatic to *agr* in this context (Beenken et al., 2010).

This accounts for our overall focus on limiting the regulatory functions of *sarA* as a means of limiting biofilm formation and thereby enhancing the therapeutic response in the context of *S. aureus* biofilm-associated infection. It also accounts for our focus on Newman in these studies in that mutation of *sarA* has a limited impact on biofilm formation in this strain by comparison to contemporary clinical isolates of *S. aureus*. The results we present demonstrate that *saeRS* and *sarA* work in concert with each other to limit the production of extracellular proteases and promote biofilm formation in *S. aureus*. Our studies employing an *sspA::lux* reporter suggest that this occurs at the transcriptional level, although it remains unknown whether this effect on proteases occurs via a direct or indirect mechanism. The production of SarA was unaffected by the functional status of *saeRS,* while expression of *saeRS* was reduced in a *sarA* mutant. This was previously reported to be the case in a COL sarA mutant [23], although it was not found to be the case in the clinical isolate UAMS-1 [24]. This suggests that this effect is strain-dependent. Nevertheless, based on this, we propose a model in which *sarA* represses the production of extracellular proteases via both *saeRS* dependent and *saeRS* independent pathways (Fig. 10). At the same time, activation of *saeRS* promotes transcription of *fnbA*. When taken together, this promotes the accumulation of critical proteins that promote biofilm formation, including FnbA and Spa. While the *saeRS*-independent pathway of *sarA*-mediated regulation has the greater overall effect, the *saeRS*-dependent pathway plays a significant role in that constitutive activation of *saeRS* can compromise the impact of *sarA* on protease production and biofilm formation. Both *sarA* and *saeRS* also modulate the production of surface adhesins at the transcriptional level, but in the absence of the reduced production of extracellular proteases owing to constitutive activation of saeRS, the phenotypic impact of this is overridden by the degradation of these adhesins due to the increased production of specific extracellular proteases, including aureolysin, SspA and/or SspB.

The accessory gene regulator (*agr*) also influences all of these phenotypes, but the impact of *agr* is opposite to that of both *sarA* and *saeRS* (Fig. 10). Additionally, expression of *sarA* influences the expression of *agr*, but mutation of *sarA* and *agr* have opposite effects on protease production, the accumulation of surface-associated adhesins, and biofilm formation [10]. This demonstrates that it is also the *agr*-independent effects of *sarA* that play the phenotypically-defining role in biofilm formation [10]. Thus, while mutation of *agr* is a common occurrence, particularly under *in vivo* conditions [25], this would not have a therapeutically relevant impact on the use of inhibitors aimed at limiting the expression and/or function of *sarA* as a means of limiting biofilm formation. In contrast, since *sarA* and *saeRS* play similar roles in biofilm formation, mutation of *saeRS* resulting in inactivation would only augment the therapeutic effect of such inhibitors.

However, the results we present also suggest that mutations that enhance the regulatory impact of *saeRS* to a degree that like observed in Newman could compromise the therapeutic utility of therapeutic strategies targeting *sarA*. This is particularly true since mutation of *sarA* in the *fnbA*-positive derivative of Newman resulted in an increased rather than decreased capacity to form a biofilm, at least when assessed using a plasmid-borne copy of *fnbA*. Nevertheless, constitutive activation of *saeRS* as observed in Newman is associated with a single point mutation [9], and this makes it imperative to determine whether the limited impact of mutating *sarA* on biofilm formation in Newman is therapeutically relevant in the context of biofilm-associated infection and, if so, to assess the frequency with which such activating mutations occur under *in vivo* conditions with the selective pressure of antibiotic therapy.

## Materials and Methods

### Bacterial Strains and Growth Conditions

The *S. aureus* strains examined in this study are listed in Table 1. Newman, its *saeS*-repaired P18L derivative, and its *saeRS* mutant were generated as previously described [8]. Experiments done with the USA300 isolate FPR3757 were done using a derivative in which the plasmid conferring resistance to erythromycin and kanamycin/neomycin was cured as previously described [8]. Mutation of *sarA*, *aur*, *fnbA*, *fnbB, spa,* and *sspABC* in these strains was done by Φ11-mediated transduction from existing mutants [10], [26]–[29]. The FPR3757 *saeRS* mutant was constructed using the pKOR1 system as previously described [30]. All strains were maintained as stock cultures at −80°C in tryptic soy broth (TSB) containing 25% (vol/vol) glycerol. For each experiment, the appropriate strains were retrieved from cold storage by plating on tryptic soy agar (TSA) with antibiotic selection. Antibiotics were used at the following concentrations: erythromycin (Erm; 5 µg per ml), tetracycline (Tet; 3 µg per ml), kanamycin (Kan; 50 µg per ml), and neomycin (Neo; 50 µg per ml).

**Table 1:** Bacterial Strains Used in This Study.

Strain	Description	Reference
**UAMS-1**	MSSA, osteomyelitis isolate	[35]
UAMS-929	UAMS-1, *sarA*::kan	[6]
UAMS-2168	UAMS-1, Δ*saeRS*	This Study
UAMS-2171	UAMS-1, Δ*saeRS*, *sarA*::kan	This Study
**UAMS-1782**	USA300, FPR3757	[10]
UAMS-1804	UAMS-1782, *sarA*::kan	[10]
UAMS-1901	UAMS-1782, *sarA*::kan, pSARA	[10]
**UAMS-1794**	UAMS-1782, Erm-sensitive	[8]
UAMS-1802	UAMS-1794, *sarA*::kan	[8]
UAMS-2258	UAMS-1794, Δ*saeRS*	This Study
UAMS-2285	UAMS-1794, Δ*saeRS*, *sarA*::kan	This Study
UAMS-3057	UAMS-1794, Δ*saeRS*, *aur*::erm	This Study
UAMS-3058	UAMS-1794, Δ*saeRS*, *sspABC*::erm	This Study
**UAMS-200**	Newman	[6]
UAMS-2167	Newman, *saeS* (P18L) (CYL11481)	[30]
UAMS-2166	Newman, Δ*saeRS* (CYL11771)	[30]
UAMS-988	Newman, *sarA*::kan	[6]
UAMS-2170	Newman, *saeS*(P18L), *sarA*::kan	[8]
UAMS-2169	Newman, Δ*saeRS*, *sarA*::kan	[8]
UAMS-2250	Newman, *sarA*::kan, *aur*::erm	This Study
UAMS-2226	Newman, *saeS*(P18L), *sarA*::kan, *aur*::erm	[8]
UAMS-190	Newman, *fnbA*::tet, *fnbB*::erm (DU5886)	[27]
UAMS-3060	Newman, *fnbA*::tet, *fnbB*::erm, *sarA*::kan	This Study
UAMS-3047	Newman, *sspA*::lux	This Study
UAMS-3045	Newman, *saeS*(P18L), *sspA*::lux	This Study
UAMS-3049	Newman, Δ*saeRS, sspA*::lux	This Study
UAMS-3048	Newman, *sarA*::kan, *sspA*::lux	This Study
UAMS-3046	Newman, *saeS*(P18L), *sarA*::kan, *sspA*::lux	This Study
UAMS-3050	Newman, Δ*saeRS*, *sarA*::kan, *sspA*::lux	This Study
UAMS-187	Newman, *spa*::tet	[28]
UAMS-3090	Newman, *spa*::tet, *sarA*::kan	This Study
UAMS-3091	Newman, *saeS*(P18L), *spa*::tet	This Study
UAMS-3092	Newman, *saeS*(P18L), *spa*::tet, *sarA*::kan	This Study
**UAMS-2227**	Newman, pFNBA	This Study
UAMS-2228	Newman, *saeS*(P18L), pFNBA	This Study
UAMS-3042	Newman, Δ*saeRS*, pFNBA	This Study
UAMS-3030	Newman, *sarA*::kan, pFNBA	This Study
UAMS-3031	Newman, *saeS*(P18L), *sarA*::kan, pFNBA	This Study
UAMS-3043	Newman, Δ*saeRS*, *sarA*::kan, pFNBA	This Study
UAMS-3051	Newman, *sarA*::kan, *aur*::erm, pFNBA	This Study
UAMS-3080	Newman, *sarA*::kan, *sspABC*::erm, pFNBA	This Study
UAMS-3052	Newman, *saeS*(P18L), *sarA*::kan, *aur*::erm, pFNBA	This Study
UAMS-3081	Newman, *saeS*(P18L), *sarA*::kan, *sspABC*::erm, pFNBA	This Study
UAMS-3067	Newman, *fnbA*::tet, *fnbB*::erm, pFNBA	This Study
UAMS-3068	Newman, *fnbA*::tet, *fnbB*::erm, *sarA*::kan, pFNBA	This Study
**Plasmid**		
Psara		[34]
pLL99		This Study
pFnbA		This Study
* sspA*::*lux*		This Study

For phenotypic assays, strains were grown in TSB supplemented with 0.5% glucose and 3.0% sodium chloride without antibiotic selection at 37°C. Biofilm formation was assessed using a static microtiter plate assay in which the substrate was first coated with plasma proteins as previously described [12]. For other assays, cultures were grown with constant aeration and a medium-to-flask volume ratio of 0.40. The post-exponential growth phase was defined as an optical density at 560 nm (OD_560_) of 3.0, while stationary-phase samples were defined by overnight (16-h) growth.

### Plasmid Construction

pLL99 was constructed by amplifying the region containing *attP1* and *attP2* from pKOR1 and cloning into pLI50 using KpnI and XbaI. To construct pFNBA, *fnbA* and its promoter region were amplified from UAMS-1 using primers that incorporated the corresponding *att* sites and cloned into pLL99 using the Gateway BP Clonase II enzyme (Invitrogen, Grand Island, NY). The *ssp::*lux reporter was generated by amplifying the promoter region of the *sspABC* operon from UAMS-1 and cloning into the EcoR1 site of pMK4 *lux ABCDE* [31]. All primers used in PCR amplifications are listed in Table 2.

**Table 2:** PCR Primers and Probes Used in This Study.

Primer	Oligonucleotide Sequence (5′→3′)
*fnbA-*attB1^1^	GGGGACAAGTTTGTACAAAAAAGCAGGCTCT GCAGAAAATCGTCTGAAATACTCAG
*fnbA*-attB2^1^	GGGGACCACTTTGTACAAGAAAGCTGGGT GAACGCCTTCATAGTGTCATTGAG
*sspA* pro S	TAATCTACCTTTGGCCAAAC
*sspA* pro AS	CCTTTCATCTAAAAACCTCC
KpnI-attP2	GGTACCCAGGAAACAGCTATGACCATGTA
XbaI-attP1	TCTAGATAGTCACGAATTCTGTAAAACGACG
*saeR*-F	CGCCTTAACTTTAGGTGCAGATGAC
*saeR*-R	ACGCATAGGGACTTCGTGACCATT
*saeR*-Probe	56-FAM/CCATCATCAACCAGTTGAACAACTGTCGT/3BHQ_1/
16S-F	TGAGATGTTGGGTTAAGTCCCGCA
16S-R	CGGTTTCGCTGCCCTTTGTATTGT
16S-Probe	AGCGCAACCCTTAAGCTTAGTTGCCA

1 Underlined sequences correspond to attB and attP sites, as indicated.

### Western Blotting

Relative amounts of protein A (Spa) were assessed by Western blot. Primary antibody was rabbit anti-Protein A (Sigma Chemical Co., St. Louis, MO) used at a 1∶4000 dilution. Secondary antibody was horseradish peroxidase (HRP)-conjugated goat anti-rabbit IgG (Sigma Chemical Co., St. Louis, MO). Western blots were developed using SuperSignal West Femto Chemiluminescent Substrate kit (Thermo Fisher Scientific, Rockford, IL). Extracellular protein A (eSpa) was assessed using standardized cell-free supernatants. Relative amounts of surface-anchored protein A were assessed using cell wall extracts prepared as previously described [32]. Briefly, cells from 1 ml of an overnight culture standardized to an OD_560_ of 14 were harvested by centrifugation at 8,000×g for 3 minutes, washed twice, and resuspended in a buffer consisting of 40 mM Tris-HCl (pH 7.5), 100 mM NaCl, 20 mM MgCl_2_, 1×protease inhibitor cocktail (Roche), 27% sucrose, 100 µg/ml lysostaphin, and 1 unit of DNase (Sigma Chemical Co., St. Louis, MO). Samples were incubated for 4 hours at 37°C before centrifuging at 6000×g for 20 min at 4°C. Samples for analysis were then collected by TCA precipitation as previously described [32].

Relative amounts of FnbA were assessed by ligand binding western blot using whole cell lysates as previously described [33]. Briefly, cells were harvested from stationary phase cultures, washed twice with water, and lysed by incubation for 20 min at 37°C in phosphate-buffered saline (PBS) containing 1 mM CaCl_2_, 0.5 mM MgCl_2_, 70 µg/ml of lysostaphin, and 2 units of DNase (Sigma Chemical Co., St. Louis, MO). Protein concentrations were determined by Bradford assay, and 6 µg of protein per sample loaded on a 3–8% Tris-Acetate SDS-PAGE gel (Invitrogen, Grand Island, NY) as previously described [34]. Proteins were transferred to PVDF membranes and blocked with 1% bovine serum albumin overnight before being incubated for 2 hours at room temperature in buffer containing with 15 µg/ml of human fibronectin (Millipore, Billerica, MA). After washing, membranes were exposed to murine IgG antibody against the N-terminus of human fibronectin (Millipore, Billerica, MA) diluted 1∶4000. Blots were then exposed to secondary antibody (horseradish peroxidase (HRP)-conjugated goat anti-mouse IgG) before development with the SuperSignal West Femto Chemiluminescent Substrate kit (Thermo Fisher Scientific, Rockford, IL). Production of SarA was also assessed using whole cell lysates as previously described [34].

### Transcriptional Analysis

To assess the levels of *saeRS* expression, total bacterial RNA was isolated using the Qiagen RNeasy mini-kit as previously described [6]. Quantitative, real-time reverse transcription-PCR (qRT-PCR) was then performed using *saeR*-specific primers and a corresponding TaqMan probe (Table 2). Results were calibrated by comparison to the results obtained with the same RNA samples using primers and a TaqMan probe corresponding to a 16S rRNA gene (Table 2). Results are reported as relative units by comparison to the results observed in the indicated strains, with the latter being set to a value of 1.0.

### Production of Extracellular Proteases

Protease activity was assessed by zymogram as previously described [10] using 10% gelatin gels (Invitrogen, Carlsbad, CA).

### Assessment of *ssp* Expression

Stationary phase (16 hour) cultures were used to inoculate 96-well white, clear-bottom plates (Corning, Lowell, MA) to an OD_560_ of 0.05. Plates were incubated at 37°C for 4.5 hours, followed by assessment of luminescence on a plate reader.

### Statistical Analysis

Statistical analysis of results comparing wild-type strains was done using the Students t-test. Statistical analysis of results comparing different strains with their isogenic *sarA* mutants was done by ANOVA based on all pair wise comparisons. In both cases ***p*** values <0.05 were considered significant.
